# Evaluation of vaccine candidates against *Rhodococcus equi* in BALB/c mice infection model: cellular and humoral immune responses

**DOI:** 10.1186/s12866-024-03408-z

**Published:** 2024-07-08

**Authors:** Lu Liu, Peng Cai, Weifang Gu, Xingxun Duan, Shiwen Gao, Xuelian Ma, Yuhui Ma, Siyuan Ma, Guoqing Li, Xiangyu Wang, Kuojun Cai, Yanfeng Wang, Tao Cai, Hongqiong Zhao

**Affiliations:** 1 College of Veterinary Medicine, Xinjiang Agricultural University, Urumqi, China; 2 Xinjiang Key Laboratory of New Drug Study and Creation for Herbivorous Animal, Urumqi, China; 3 Zhaosu Xiyu Horse Industry Co., Ltd., Yining, China; 4 Xinjiang Agricultural Vocational Technical College, Changji, China

**Keywords:** *Rhodococcus equi*, Vaccine candidate, Recombinant protein, Humoral immunity, Cellular immunity

## Abstract

*Rhodococcus equi* (*R. equi*) is a zoonotic opportunistic pathogen that mainly causes fatal lung and extrapulmonary abscesses in foals and immunocompromised individuals. To date, no commercial vaccine against *R. equi* exists. We previously screened all potential vaccine candidates from the complete genome of *R. equi* using a reverse vaccinology approach. Five of these candidates, namely ABC transporter substrate-binding protein (ABC transporter), penicillin-binding protein 2 (PBD2), NlpC/P60 family protein (NlpC/P60), esterase family protein (Esterase), and M23 family metallopeptidase (M23) were selected for the evaluation of immunogenicity and immunoprotective effects in BALB/c mice model challenged with *R. equi*. The results showed that all five vaccine candidate-immunized mice experienced a significant increase in spleen antigen-specific IFN-γ- and TNF-α-positive CD4 + and CD8 + T lymphocytes and generated robust Th1- and Th2-type immune responses and antibody responses. Two weeks after the *R. equi* challenge, immunization with the five vaccine candidates reduced the bacterial load in the lungs and improved the pathological damage to the lungs and livers compared with those in the control group. NlpC/P60, Esterase, and M23 were more effective than the ABC transporter and PBD2 in inducing protective immunity against *R. equi* challenge in mice. In addition, these vaccine candidates have the potential to induce T lymphocyte memory immune responses in mice. In summary, these antigens are effective candidates for the development of protective vaccines against *R. equi*. The *R. equi* antigen library has been expanded and provides new ideas for the development of multivalent vaccines.

**Supplementary Information:**

The online version contains supplementary material available at 10.1186/s12866-024-03408-z.

## Introduction

*Rhodococcus equi* (*R. equi*) is a zoonotic opportunistic pathogen that mainly causes fatal lung and extrapulmonary abscesses in foals and immunocompromised individuals and threatens the health of the livestock industry and public health safety worldwide [1–3]. Although *R. equi* affects a variety of animal species, *R. equi* is a well-known major horse pathogen in veterinary medicine, causing life-threatening multifocal pneumonia in foals with frequent extrapulmonary involvement. Attack rates in horse-breeding farms where the disease caused by *R. equi* is endemic are typically 20–40% [3, 4]. In humans, *R. equi* mainly causes pneumonia that radiographically and pathologically resembles pulmonary tuberculosis. In recent years, *R. equi* has become more widely recognized due to the growing number of human cases [3, 5–8].

The standard treatment for *R. equi* infection in foals combines macrolides (erythromycin, clarithromycin, or azithromycin) and rifampicin [9]. With the widespread use of these antibiotics, the emergence and rapid evolution of multidrug-resistant (MDR) isolates have been reported in the USA, China, Poland, and other countries [10–12]. Vaccines are an effective strategy against MDR pathogens, but currently, no licensed vaccines against *R. equi* exist. Oral administration (gavage) of live virulent *R. equi* has been shown to protect foals against severe *R. equi* challenges [13, 14]. However, the use of live virulent pathogens as vaccines is not permitted due to risks to the environment and host. The inactivated vaccine ensures the structural integrity of *R. equi* and can induce humoral and cellular immune responses in foals, but it is not effective in protecting foals from the challenge of virulent *R. equi* [15, 16]. To date, the only licenced approach to reduce the incidence and severity of *R. equi* pneumonia is prophylactic transfusion of *R. equi*-specific hyperimmune plasma, but the results of this approach in experimental and field studies are conflicting [17, 18]. Indeed, similar to *Mycobacterium tuberculosis* (*M. tb*), the cellular immune response against this intracellular bacterium pathogen is largely thought to exert major immune protection, although the antibody response also mediates immune protection [19, 20]. Cellular immunity is the basis of host responses against *R. equi* infection. In this context, antigen-based subunit vaccines may be safer and effective options. However, only a limited number of *R. equi* antigens have been reported and validated, among which virulence-associated proteins (Vaps) have been widely investigated in vaccine development to prevent *R. equi* infections. Vaps-based recombinant protein subunit vaccines, recombinant DNA vaccines, vector vaccines, and other engineered vaccines can induce specific humoral and cellular immune responses in the host to varying degrees, but they provide inadequate protection for foals [21–23]. Bacteria have a complete cellular structure, and the complexity of their composition makes it theoretically impossible for a single antigen to be better than inactivated or live attenuated vaccines. Therefore, screening and identifying more antigens and developing multitargeted vaccines against *R. equi* infection may be a safer and effective strategy.

Reverse vaccinology is a powerful approach for screening vaccine targets. This approach is based on the pathogenic genome sequence and uses a series of bioinformatics tools to predict pathogen virulence factors, essential proteins, membrane surface proteins and extracellular proteins and to evaluate the antigenicity, physicochemical properties and toxicity of proteins [24]. This approach can be used to efficiently screen antigens for the development of vaccines without cultivating pathogens, overcoming the limitations of traditional vaccine development methods. Reverse vaccinology has been extensively used for the screening of vaccine candidates for various pathogens, such as *serogroup B Neisseria meningitidis* (*menB*) [25], *Acinetobacter baumannii* [26], *Brucella* spp. [27], *Shigella dysenteriae* [28], and *Mycoplasma synoviae* [29]. With this approach, *menB* vaccines have been successfully developed [25]. In a previous report, we screened conserved core proteins from the complete genomes of 16 *R. equi* isolates from different hosts in different countries. Then, we used reverse vaccinology strategies to identify 12 vaccine candidates from the core protein pool based on host homology, subcellular localization, antigenicity, transmembrane helices, physicochemical properties, immunogenicity, and virulence factor/antigen database alignment [30]. Here, five vaccine candidates, namely, ABC transporter substrate-binding protein (ABC transporter), penicillin-binding protein 2 (PBD2), NlpC/P60 family protein (NlpC/P60), esterase family protein (Esterase), and M23 family metallopeptidase (M23), were selected, and their potential to induce protection immune against challenges by *R. equi* in mice was further evaluated.

Notably, equine infection models have also been used for the development of *R. equi* vaccines [31, 32]. However, using horses for in vivo studies with *R. equi* poses obvious economic and logistical constraints, and BALB/c mice have been developed as models to investigate *R. equi* infections and vaccine development [33, 34]. Therefore, the focus of this study was to validate the efficacy of five vaccine candidates in inducing antigen-specific immune responses and protection against challenges by *R. equi*. This study aimed to establish an antigen library for the development of an *R. equi* vaccine, which can be gradually refined it in future work.

## Methods

### Bacterial strains and animals

The *R. equi* 103S strain was kindly provided by Prof. Haixia Luo (College of Life Sciences, Ningxia University, Ningxia, China). Six-week-old female BALB/c mice were purchased from the Laboratory Animal Center, Xinjiang Medical University (Urumqi, China). The mice were acclimatized for 1 week before the start of the experiment and were housed at a temperature of 22 ~ 25 °C under a 12/12 h light/dark cycle with food and water available ad libitum. In animal experiments, every effort was made to minimize the pain and distress of the animals. All experimental procedures were carried out strictly according to the guidelines for the euthanasia use of laboratory animals (GB/T 39760 − 2021), and approved by the Animal Ethics Committee of Xinjiang Agricultural University, Urumqi, China (Ethical Committee Approval number 2022013).

### Expression and purification of recombinant antigenic proteins

The recombinant ABC transporter, PBD2, NlpC/P60, Esterase, and M23_pET-30a(+) plasmids were constructed by the GenScript Biotechnology Co., Ltd. (Nanjing, China). All proteins contained a His-tag at the N-terminus (MHHHHHH-). BL21(DE3) cells containing the recombinant plasmid were cultured in LB medium supplemented with 30 µg/ml kanamycin at 37 °C at 180 rpm to an OD_600 nm_ of 0.6 (~ 3 h). Cultures were induced with 1.0 mM IPTG for 5 h at 37 °C. After induction, the *E. coli* cells were centrifuged at 4,146 × g for 10 min at 4 °C (MIKRO220R, Hettich, Germany). The *E. coli* pellet was resuspended in PBS and sonicated (power 45%, 2 s on, 3 s off, 10 min; JY92-IIN, SCIENTZ, Ningbo, China). Then, the cell suspensions were collected by centrifugation at 4 °C and 4,146 × g for 20 min. The cell suspensions containing inclusion bodies were dissolved in 8 M urea buffer at 4 ℃ for 20 min. The samples were filtered through a 0.45 μm filter and loaded onto affinity chromatography columns (FCL06, Beyotime, Beijing, China) loaded with Ni-nitrilotriacetic acid (Ni-NTA) resin (BBI, Shanghai, China). Each target protein was eluted with elution buffer (0.5 M NaCl, 50 mM Tris-HCl, and 600 mM imidazole, pH of 8.0). Then, each purified protein was dialyzed in buffers containing 6 M, 4 M, and 2 M urea, respectively, for 6 h. Finally, the dialysis bag was placed in PBS for dialysis overnight. The refolded protein was collected and then concentrated using Millipore Amicon^®^ Ultra Ultrafiltration tubes. The protein concentration was determined using a bicinchoninic acid protein assay kit (P0012, Beyotime, Shanghai, China). The purity of the recombinant protein was determined by SDS-PAGE and Coomassie blue staining. The purified protein was subjected to removal with a ToxinEraser™ endotoxin removal kit (L00338, GenScript, Nanjing, China) to remove endotoxins and stored at -80 °C until use.

### Immunization and *R. equi* 103S challenge procedure

One hundred forty BALB/c mice were separated into seven groups and immunized three times at two-week intervals (weeks 0, 2, and 4). The blank control group (twenty mice) and the infection control group (twenty mice) were administered adjuvant alone in PBS at an adjuvant volume ratio of 1:1 in a 200 µL volume (multispot subcutaneous injection in the back). The ABC transporter, PBD2, NlpC/P60, Esterase, and M23 groups (*n* = 20 mice/group) were primed with 100 µg of recombinant proteins combined with Freund’s complete adjuvant (F5881, Sigma, Shanghai, China) at a protein to adjuvant volume ratio of 1:1 and boosted with 50 µg of recombinant proteins combined with Freund’s incomplete adjuvant (F5506, Sigma, Shanghai, China) at a protein to adjuvant volume ratio of 1:1 in a 200 µL volume (multispot subcutaneous injection in the back). Blood samples were collected from the tails of the mice weekly after immunization. Two weeks after the last immunization, five mice in each group were euthanized (intraperitoneal injection of 200 mg/kg sodium pentobarbital), splenocytes and serum samples were collected, and immunogenicity was analysed by flow cytometry (FCM) and enzyme-linked immunosorbent assays (ELISAs).

Two weeks after the last immunization, the remaining mice in the infection control, ABC transporter, PBD2, NlpC/P60, Esterase, and M23 groups (*n* = 15 mice/group) were challenged with sublethal doses (2.34 × 10^7^ CFU/mouse) of *R. equi* 103S by intraperitoneal injection (the sublethal dose was determined by a pre-experiment, Table S1 and Fig. S1), and the blank control group was challenged with PBS. Feed intake was monitored daily for 10 days. Body weight was monitored weekly for 4 weeks. Blood samples were collected from the tails of the mice every week after the challenge, and serum was isolated for the detection of antibodies and cytokines. At 1, 2, and 4 weeks after the challenge, five mice were randomly selected from each group for euthanasia. Lungs and livers were collected for macroscopic pathological, histopathological, and bacterial load analysis. Spleens were collected, and splenocytes were isolated for analysis of antigen-specific TNF-α- and IFN-γ-positive CD4 + and CD8 + T lymphocytes.

### ELISA for serum antibody response

Serum antigen-specific antibody levels were monitored by ELISA. The 96-well high-binding ELISA plates (FST015, Beyotime, Shanghai, China) were coated with the ABC transporter, PBD2, NlpC/P60, Esterase, and M23 proteins (20 ng/100 µL/well) in coating buffer (C1055, Solarbio, Beijing, China) at 4 °C overnight. The plates were then blocked with 1% BSA-PBST (100 µL/well) at 37 °C for 2 h. After five washes with PBST, serial dilutions of serum were added and incubated at 37 °C for 1.5 h. After washing, horseradish peroxidase (HRP)-conjugated goat anti-mouse IgG (1:5,000, Proteintech, Wuhan, China), IgM (1:5,000, bs-0368G, Bioss, Beijing, China), and IgG1 (MS3211)/IgG2a (MS3221) (1:10,000, PhosphoSolutions, Denver, USA) were added and incubated at 37 °C for 2 h. All antibodies were diluted in 1% BSA-PBST. Finally, TMB substrate (100 µL/well, T0440, Sigma-Aldrich) was added and incubated at 37 °C for 15 min for colour development. The readout was recorded at OD_450 nm_ using a microplate reader (SynergyHTX, BioTek Instruments, Winooski, VT, USA) after 2 N of sulfuric acid was added to stop the reaction. The serum antibody titre was the largest dilution at which the OD_450 nm positive_/OD_450 nm negative_ was > 2.1.

### FCM for antigen-specific TNF-α- and IFN-γ-positive T lymphocytes

At 1, 2, and 4 weeks after *R. equi* 103S challenge, spleens were collected to isolate splenocytes. Antigen-specific TNF-α- and IFN-γ-positive CD4 + and CD8 + T lymphocytes in splenocytes were evaluated using FCM. Approximately 3.0 × 10^6^ splenocytes were seeded in 6-well cell culture plates and stimulated with 10 µg/mL ABC transporter, PBD2, NlpC/P60, Esterase, or M23 recombinant protein for 16 h at 37 °C. After 16 h of stimulation, breamectin A (10 µg/mL, 423303, Biolegend, California, USA) was added and incubated for 6 h. The cells were collected and stained with PerCP-conjugated anti-mouse CD4 (100431, Biolegend) and FITC-conjugated anti-mouse CD8a (100803, Biolegend) antibodies for 30 min at 4 °C in the dark. The cells were washed twice with cold FCM staining buffer (MB-089-0500, Rockland, Limerick, Ireland), fixed and permeabilized with FIX&PERM solution (GAS-002 M, Nordic Mubio) for 20 min, and then washed with perm wash buffer. Then, the cells were stained with APC-conjugated anti-mouse IFN-γ (505809, Biolegend) and PE-conjugated anti-mouse TNF-α (506305, Biolegend) at 4 °C for 20 min in the dark. The cells were washed twice with perm wash buffer, resuspended in staining buffer, visualized, and analysed using FCM (S3e, Bio-Rad, California, USA). Cells subjected to isotype-matched IgG staining were included as controls.

### ELISA for serum cytokine secretion

Serum samples were collected at 2 weeks after the last immunization and 1, 2, and 4 weeks after the *R. equi* 103S challenge, after which the levels of cytokines were measured. Commercial IFN-γ (E-EL-M0048c), TNF-α (E-EL-M3063), IL-4 (E-EL-M0043c), and IL-17 A (E-EL-M0047c) mouse ELISA kits (Elabscience, Wuhan, China) were used to detect the serum cytokines, and a standard curve was generated according to the manufacturers’ instructions.

### Bacterial load in the lungs

At 1, 2, and 4 weeks after *R. equi* 103S challenge, the lungs were collected under sterile conditions and then washed with sterile PBS. The tissue was homogenized in sterile PBS (g tissue/mL PBS) using a Tissuelyser (F6/10, Jingxin, Shanghai, China), and 10-fold serial dilutions were prepared to 10^− 4^. The diluted tissue homogenates were coated on TSA plates and incubated at 37 °C for approximately 30 h before counting. CFU data were log-transformed before analyses.

### Histopathology

Unfixed lungs and livers were photographed with a smartphone (iPhone 14 Pro) for macropathology analysis. Subsequently, the lungs and livers were fixed in 4% paraformaldehyde, dehydrated with graded ethyl alcohol, cleared with xylene, embedded in paraffin, and sliced into 4-µm-thick sections. Then, the sections were stained with haematoxylin and eosin (H&E) for histopathological examination. Slides were imaged at 40×, 200×, and 400× magnification with a Motic BA210 digital microscope (Xiamen, China) equipped with a digital camera and Motic Images Plus 3.1. H&E-stained tissue sections were scored for lung and liver histopathology. For the lung histopathological score, each slice was scored according to three classifications: widening of the alveolar septum, severity of fibrotic exudation in the alveolar space, and degree of inflammatory cell infiltration. Each classification was scored from 0 to 4 using the following scale: 0 = normal; 1 = slightly; 2 = moderately; 3 = greatly; and 4 = very seriously. For the liver histopathological score, each slice was scored according to three classifications: the severity of hepatic cord structural disorders, the severity of hepatocellular degeneration, and the degree of inflammatory cell infiltration. Each classification was scored from 0 to 4 using the following scale: 0 = normal; 1 = slightly; 2 = moderately; 3 = greatly; and 4 = very seriously.

### Statistical analysis

The data were analysed using GraphPad Prism v.5.0 (San Diego, CA), and the quantitative data are presented as the mean ± SD. One-way ANOVA followed by Tukey’s comparison test was used to analyse the statistical significance of differences. *P* < 0.05 was considered to indicate statistical significance. * *P* < 0.05; ** *P* < 0.01.

## Results

### Expression and purification of recombinant vaccine candidates

**Fig. 1:**
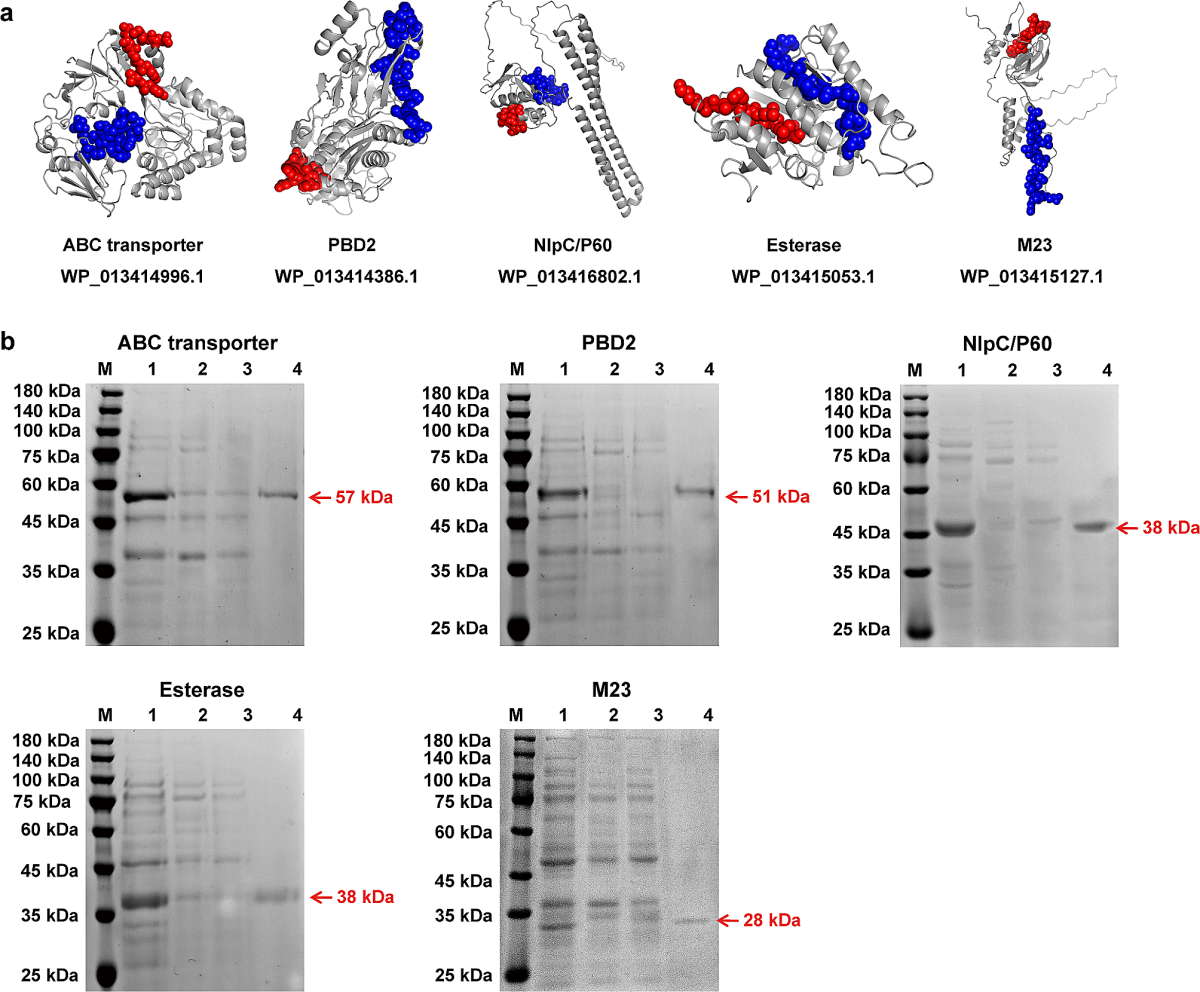
Expression and purification of the recombinant vaccine candidates. **(a)** 3D structures and epitope mapping of the vaccine candidates. **(b)** Purification of a recombinant ABC transporter (~ 57 kDa), PBD2 (~ 51 kDa), NlpC/P60 (~ 38 kDa), Esterase (38 ~ kDa), and M23 (~ 28 kDa) protein. Line M, 180 kDa protein marker; Line 1, protein after lysis in 8 M urea buffer; Line 2, flow-through from the resin; Line 3, wash-through from the resin by 100 mM imidazole; Line 4, purified protein eluted by 600 mM imidazole

A reverse vaccinological strategy was used to screen potential vaccine candidates, and five antigen proteins involved in different families, including ABC transporter, PBD2, NlpC/P60, Esterase, and M23, were selected for preliminary evaluation. More than 98% homology existed in the genomes of 16 *R. equi* strains from different countries and hosts (Table S2-S5). The open reading frame (ORF) lengths of the ABC transporter, PBD2, NlpC/P60, Esterase, and M23 were 1545 bp, 1425 bp, 1182 bp, 1011 bp, and 861 bp, encoding 537 aa, 495 aa, 384 aa, 361 aa, and 277 aa, respectively, with predicted molecular masses of ~ 57 kDa, ~ 51 kDa, ~ 38 kDa, ~ 38 kDa, and ~ 28 kDa, respectively. A supplementary file shows the sequences of the recombinant proteins and other information in more detail (Supplementary Sequence). The 3D structures of the five vaccine candidates are shown in Fig. 1A. The SDS-PAGE results showed that the recombinant proteins with the expected molecular weights were obtained after expression in BL21(DE3) cells and purification by Ni-nitrilotriacetic acid (Fig. 1B).

### Immunogenicity of vaccine candidates in BALB/c mice

**Fig. 2:**
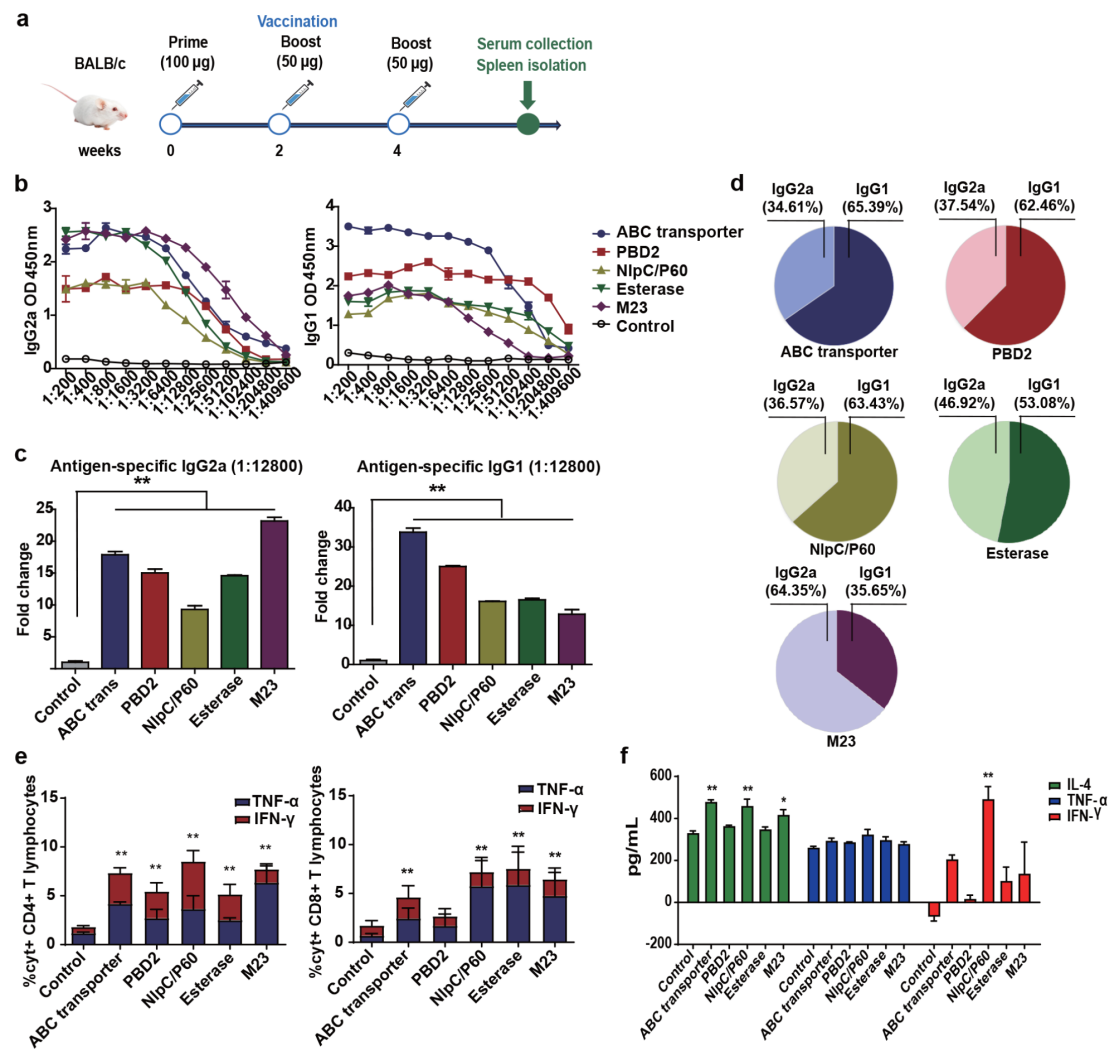
Evaluation of the immune response induced by vaccine candidates in BALB/C mice. **(a)** Schematic diagram of the study design. BALB/c mice were subcutaneously immunized with Freund’s adjuvant-formulated ABC transporter, PBD2, NlpC/P60, Esterase, or M23 recombinant protein three times at two-week intervals. The mice were euthanized two weeks after the last immunization, and blood and spleen samples were collected. **(b)** Antigen-specific IgG2a and IgG1 antibody titres. **(c)** Fold changes in IgG1 and IgG2a levels between the recombinant protein subunit vaccine immunization group and the control group. **(d)** The ratio of IgG1 to IgG2a in serum. **(e)** The proportions of IFN-γ- and TNF-α-positive CD4 + and CD8 + T lymphocytes in the splenocyte population two weeks after the last immunization. **(f)** IL-4, TNF-α and IFN-γ levels were measured using ELISA. The mean ± SD and one-way ANOVA were used to analyse the statistical significance; **(c)**, **(e)**, and **(f)** are the vaccination groups compared to the control group; * *P* < 0.05, ** *P* < 0.01

To investigate the immunogenicity of the five vaccine candidates, BALB/c mice were immunized subcutaneously with recombinant proteins mixed with Freund’s adjuvant three times (weeks 0, 2, and 4) as shown in the schematic diagram (Fig. 2A). The control group received adjuvant alone. Two weeks after the last immunization, five mice in each group were euthanized, and antigen-specific antibody levels, cytokine levels, and IFN-γ- and TNF-α-positive T lymphocyte immune responses were evaluated (Fig. 2A). The serum concentrations of antigen-specific IgG1 and IgG2a antibodies were determined using ELISA. After the last vaccination, all five candidates stimulated the production of high levels of antigen-specific IgG1 and IgG2a antibodies (Fig. 2B, C). In addition, balanced IgG1/IgG2a responses were observed in the ABC transporter- and PBD2-immunized groups. NlpC/P60, Esterase, and M23 induced high levels of IgG2a-biased responses (Fig. 2D). The antigen-specific CD4 + and CD8 + T lymphocyte reactions in the spleen were evaluated through FCM, and the gating strategy for data analysis is shown in more detail in the attached file (Fig. S2). In splenocytes, the proportions of IFN-γ- and TNF-α-positive CD4 + T lymphocytes in mice immunized with vaccine candidates were significantly greater after 16 h of in vitro stimulation with the ABC transporter, PBD2, NlpC/P60, Esterase, or M23. Additionally, mice immunized with vaccines produced significantly greater frequencies of splenic CD8 + T lymphocyte responses to the ABC transporter, NlpC/P60, Esterase, and M23 but not to PBD2 (Fig. 2E). The levels of the serum cytokine IL-4 in mice immunized with the ABC transporter, NlpC/P60 and M23, and of IFN-γ in mice immunized with NlpC/P60 were greater than those in mice immunized with adjuvant alone, while there was no difference in TNF-α levels was found between the groups. No cytokine IL-17 was detected (Fig. 2F). These data suggest that these subunit vaccines induced Th1 and Th2 cellular immune responses but did not induce a Th17 response. Overall, immunization with the five recombinant protein subunit vaccines can induce cellular and humoral immune responses in BALB/c mice.

### Recombinant protein subunit vaccines protect BALB/c mice against *R. Equi*

**Fig. 3:**
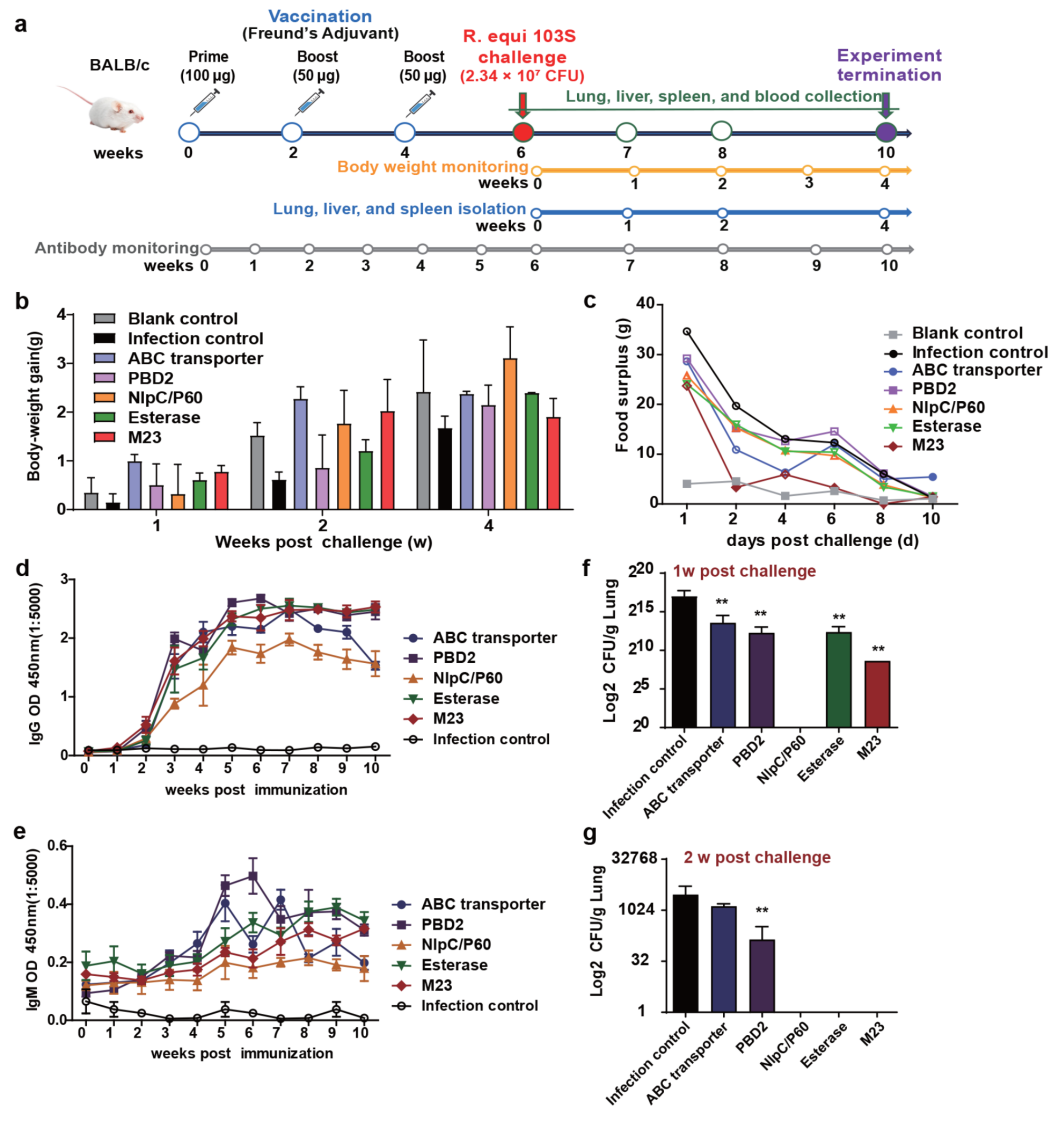
Recombinant protein subunit vaccines protect BALB/c mice against intraperitoneal challenge with *R. equi*103S. **(a)** Schematic diagram of the recombinant protein subunit vaccine immunization and *R. equi* 103 S challenge process. BALB/c mice were immunized three times (two weeks apart) with ABC transporter, PBD2, NlpC/P60, Esterase, and M23 recombinant protein subunit vaccines formulated in Freund’s adjuvant. Two weeks after the last immunization, the mice in the vaccination group were challenged with 2.34 × 10^7^ CFU of *R. equi* 103 S by intraperitoneal injection. Mice were immunized with adjuvant alone as a blank control, and mice that had been intraperitoneally challenged with *R. equi* 103 S after immunization with adjuvant alone were used as the infection control. **(b)** Body weight was measured at 1, 2, and 4 weeks after *R. equi* 103 S challenge. **(c)** Feed intake was measured daily for 10 days after the *R. equi* 103 S challenge. Serum was collected weekly for 10 weeks after immunization, and antigen-specific IgG **(d)** and IgM **(e)** antibody levels were detected by ELISA. The lung bacterial burden in mice at 1 (**f**) and 2 (**g**) weeks post-challenge was assessed. The mean ± SD and one-way ANOVA were used to analyse the statistical significance; **(f)** and **(g)** are compared with the infection control group; * *P* < 0.05, ** *P* < 0.01

Two weeks after the last immunization, the vaccinated mice were challenged by intraperitoneal injection of 2.43 × 10^7^ CFU of *R. equi* 103S per mouse and euthanized at weeks 1, 2, and 4 after the challenge to evaluate protective immunity (Fig. 3A). The body weights were monitored at 1, 2, and 4 weeks post-challenge. The body weight gains of the five vaccine candidate-immunized mice were greater than those of adjuvant-only vaccinated mice at weeks 1, 2, and 4 following *R. equi* 130 S challenge, but the differences were not statistically significant (Fig. 3B). The feed intake of the mice in all groups returned to normal at 10 days after the *R. equi* 103S challenge (Fig. 3C). Serum antibody levels were measured weekly for 10 weeks after immunization, and the results showed that mice immunized with the five subunit vaccines exhibited high levels of antigen-specific IgG and IgM. Notably, the IgG antibody titres remained high until the end of the experiment (Fig. 3D, E). The rapid clearance of *R. equi* from the lungs of challenged mice is a key hallmark of an effective vaccine. To assess this, the bacterial burdens in the lungs were measured at 1 and 2 weeks post-challenge. The bacterial burdens were significantly lower in the five vaccine candidate-vaccinated mice than in the infection control group mice at 1 week post-challenge (Fig. 3F). Remarkably, no bacteria were cultured from the lungs at 2 weeks post-challenge in the NlpC/P60-, Esterase-, or M23-vaccinated mice (Fig. 3G).

**Fig. 4:**
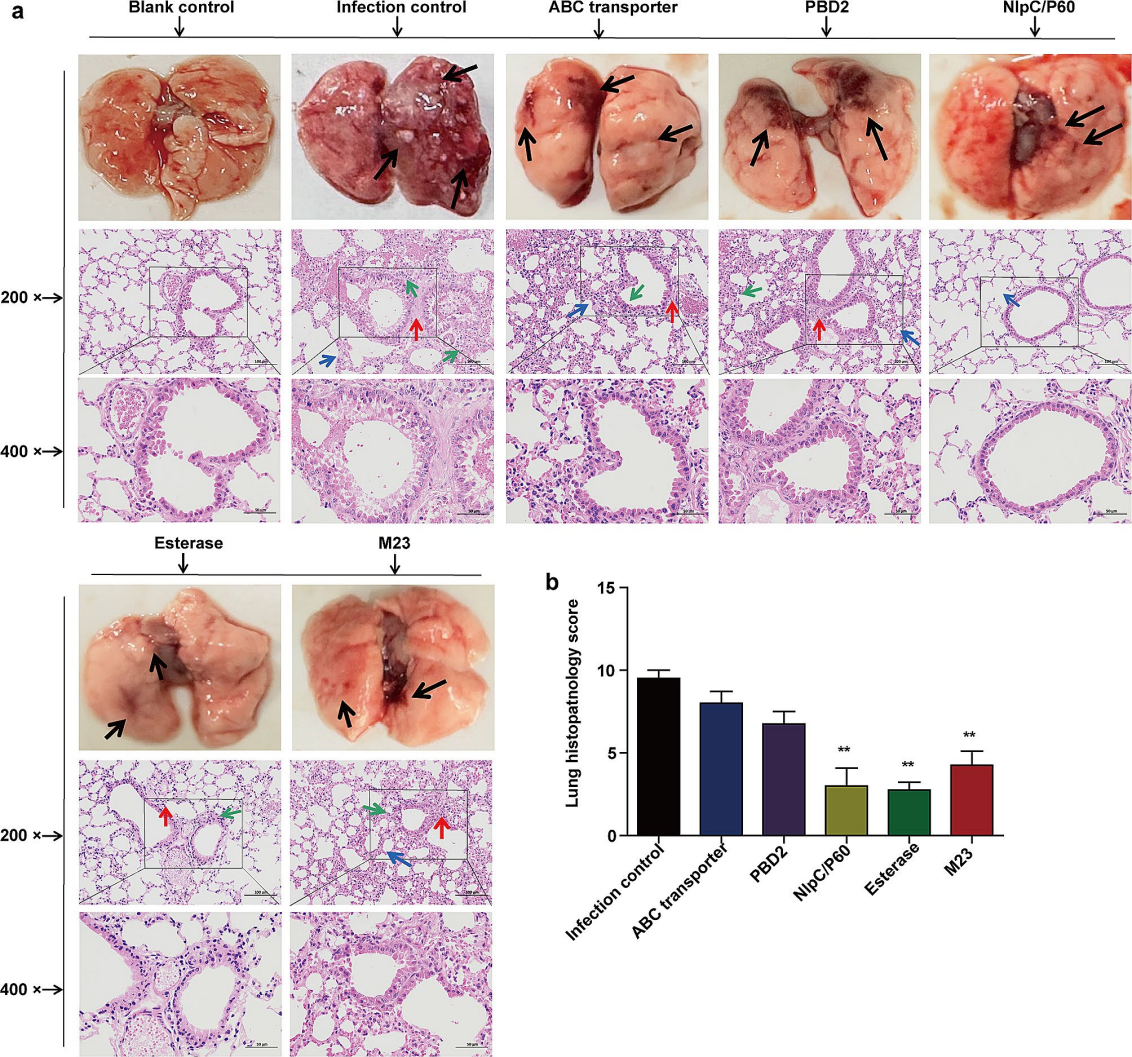
Macropathological and histopathological findings of the lungs. To evaluate the macropathology and histopathology, the lungs of mice challenged with *R. equi* 103S were photographed and stained with H&E at 2 weeks post-challenge. **(a)** Macropathological changes in the lungs: granulomas, hyperaemia, and oedema (black arrow). Histopathology changes in the lungs (scale bars, 100 μm (top) and 50 μm (bottom)): the bottom images are enlarged from the outlined areas of the top images. Intraalveolar exudation (red arrow), alveolar septum widening and rupture (blue arrow), and inflammatory cell infiltration (green arrow). **(b)** Lung histopathology score. For the histopathological score, each slice was scored according to three classifications: widening of the alveolar septum, severity of fibrotic exudation in the alveolar space, and degree of inflammatory cell infiltration. Each classification was scored from 0–4 using the following scale: 0 = normal; 1 = slightly; 2 = moderately; 3 = greatly; and 4 = very seriously. The mean ± SD and one-way ANOVA were used to analyse the statistical significance, * *P* < 0.05, ** *P* < 0.01

Macropathology was conducted on the lungs 2 weeks after the *R. equi* 103S challenge. Numerous granulomas (black arrow) were observed on the lung surfaces of the infection control mice. Large areas of congestion and oedema (black arrow) were observed in the lungs of mice immunized with ABC transporter and PBD2. In contrast, only minor lung lesions were observed in the NlpC/P60, Esterase, and M23 groups (Fig. 4A). Next, we analysed fibrotic exudation, alveolar septal changes, and inflammatory infiltration in the lung tissue by histopathology to determine whether the subunit vaccines provided protection against lung infection and injury. Mice from the infection control group displayed granulomatous pneumonia, characterized by granuloma on the lung surface (black arrow), thickened alveolar septa (blue arrow), inflammatory cell infiltration (green arrow), and fibrotic exudation in a large area of the alveolar space (red arrow). In contrast, the severity of lesions in the lung tissue of the mice vaccinated with the five subunit vaccines was reduced (Fig. 4A, B). Among them, mice in the infection control group had significantly greater lesion scores than those in the NlpC/P60, Esterase, and M23 subunit vaccine groups (Fig. 4B).

**Fig. 5:**
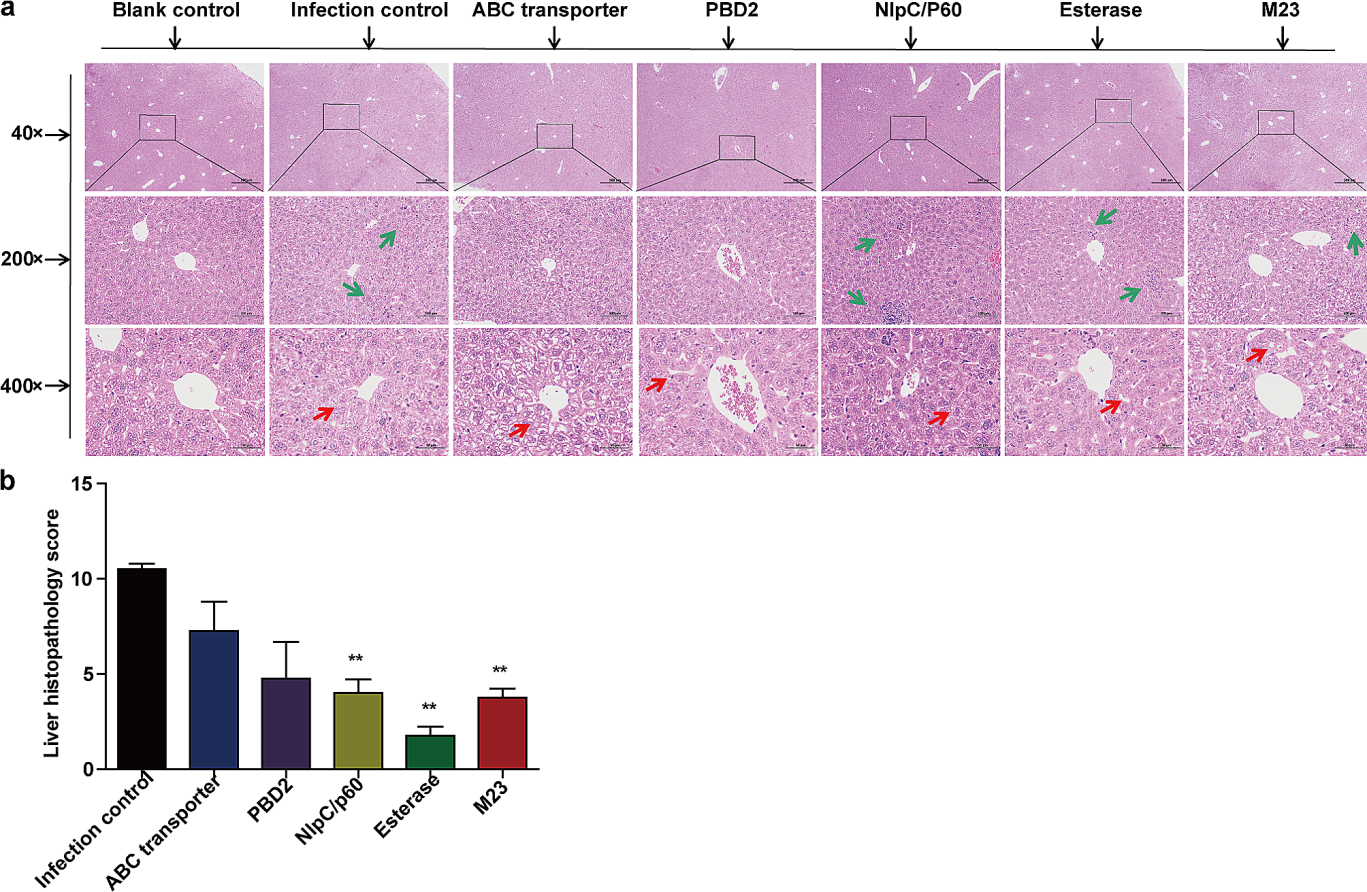
Histopathological findings in the liver. Two weeks after *R. equi* 103S challenge, histopathological changes were evaluated in livers stained with H&E. **(a)** Histopathological changes in the livers (scale bars, 500 μm (top), 100 μm (middle), and 50 μm (bottom)): the middle images are enlarged from the outlined areas of the top images. Hepatocellular degeneration (red arrow); inflammatory cell infiltration (green arrow). **(b)** For the histopathological score, each slice was scored according to three classifications: the severity of hepatic cord structural disorders, the severity of hepatocellular degeneration, and the degree of inflammatory cell infiltration. Each classification was scored from 0–4 using the following scale: 0 = normal; 1 = slightly; 2 = moderately; 3 = greatly; and 4 = very seriously. The mean ± SD and one-way ANOVA were used to analyse the statistical significance, *P < 0.05, **P < 0.01

The livers were fixed and sectioned for histopathological analysis. Five mice from the infection control group displayed a disordered hepatic cord structure, inflammatory cell infiltration (green arrow), and hepatocellular degeneration in a large area (red arrow). In contrast, the severity of the lesions in the liver tissues of PBD2, NlpC/P60, Esterase, and M23 protein-immunized mice was significantly reduced (Fig. 5A, B).

### Recombinant protein subunit vaccines induce immunity in long-term memory T lymphocytes

**Fig. 6:**
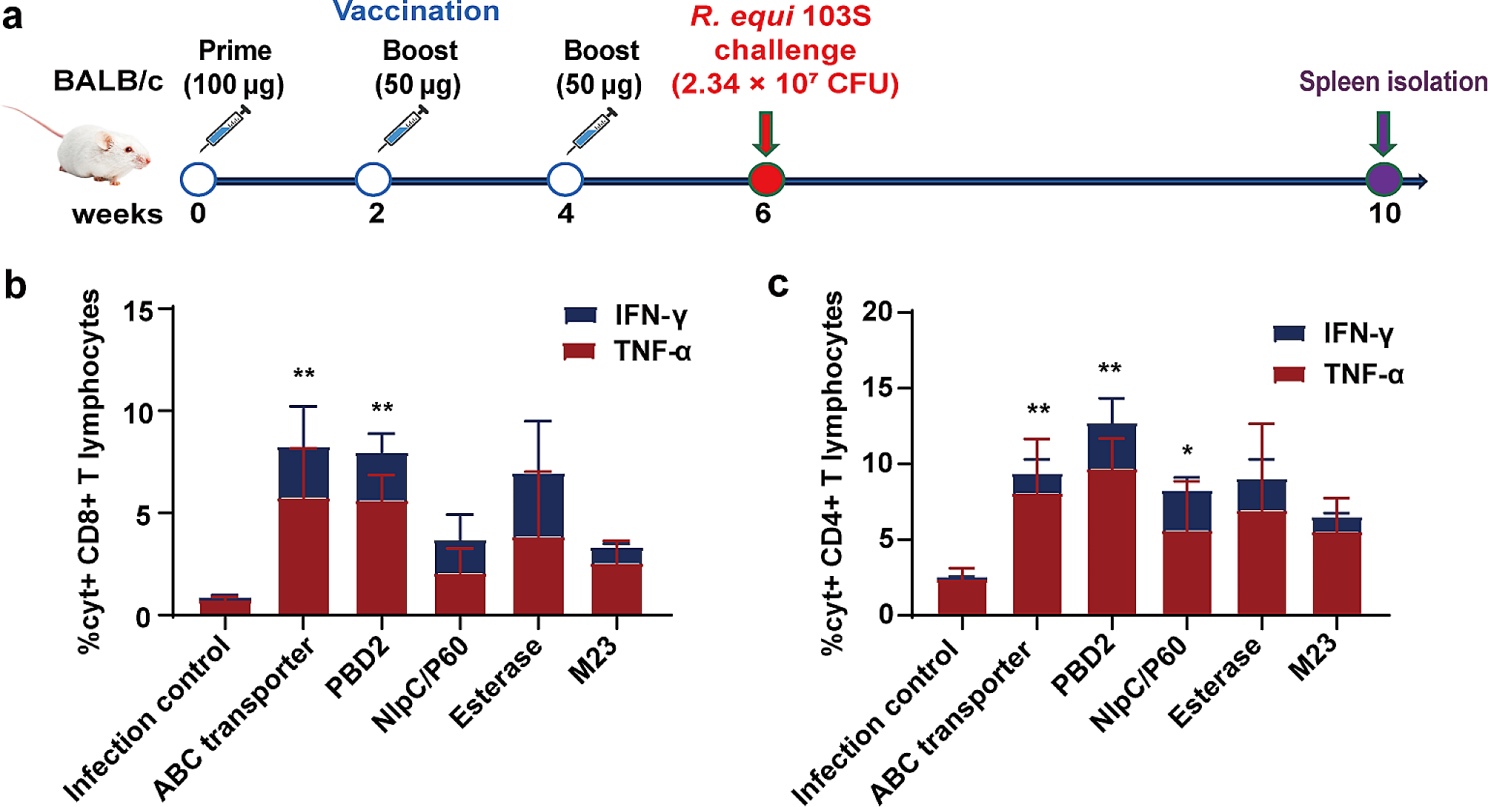
Recombinant protein subunit vaccines induce long-term memory T lymphocyte immune responses. **(a)** Schematic diagram of the vaccination, *R. equi* 103S challenge, and sample collection timeline. Ten weeks after the first immunization, the percentages of TNF-α- and IFN-γ-positive splenic CD8+ **(b)** and CD4+ (**C**) T lymphocytes were measured by FCM after restimulation with the ABC transporter, PBD2, NlpC/P60, Esterase, or M23 protein. The mean ± SD and one-way ANOVA were used to analyse the statistical significance, * *P* < 0.05, ** *P* < 0.01

Ten weeks after the first immunization, five mice in each group were euthanized. Splenocytes were isolated and stimulated with ABC transporter, PBD2, NlpC/P60, Esterase, and M23 proteins for 16 h, and long-term memory T lymphocyte immune responses were evaluated by FCM (Fig. 6A). Compared with those in the control group, the proportions of TNF-α- and IFN-γ-positive CD8 + T lymphocytes in the splenocyte population were significantly greater after restimulation with the ABC transporter and PBD2 proteins (Fig. 6B). In addition, TNF-α- and IFN-γ-positive CD4 + T lymphocytes were significantly increased after restimulation with the ABC transporter, PBD2, and NlpC/P60 proteins (Fig. 6C). These results indicate that the ABC transporter, PBD2, and NlpC/P60 recombinant protein subunit vaccines have the potential to induce long-term memory immune responses in mice.

## Discussion

*R. equi* is an important opportunistic pathogen infecting foals and has been identified as one of the most relevant antimicrobial-resistant bacteria for horses by the european food safety authority [33]. In recent years, many studies have attempted to develop effective and safer *R. equi* vaccines. The difficulty in developing a vaccine against *R. equi* is attributed to the lack of knowledge about its antigens. To date, only Vaps family proteins have shown promise as vaccine candidates, but they require further development [21–23]. For bacterial infections, a single-antigen engineered vaccine may also not be sufficient to provide effective immune protection to foals. The identification of more antigens is very important and urgent for the development of safe and effective vaccine candidates against *R. equi*.

To address this issue, we adopted a reverse vaccinology strategy from previous studies using a variety of bioinformatics tools to screen all potential vaccine candidates from the *R. equi* genome [30]. However, antigens predicted and screened based on bioinformatics tools need to be further validated by animal experiments. Therefore, in the current study, we expressed and purified five of these vaccine candidates and tested their immunogenicity and protective capacity. The results showed that all five vaccine candidate-immunized mice produced strong Th1 and Th2 immune responses and strong antibody responses. Cellular immunity plays a key role in the clearance and immunity of intracellular pathogens [34, 35]. In mice, while both CD4 + and CD8 + T lymphocytes contribute to host defence against *R. equi*, CD4 + helper T lymphocytes play a major role in the clearance of virulent *R. equi* [23, 36–39]. Foals infected with *R. equi* produce a mixed Th1/Th2 immune response [37, 40]. The activity of Th1 cytokines is essential for the elimination of intracellular pathogens. Studies have shown that immunocompetent BALB/c mice infected with virulent *R. equi* produce a Th1 cytokine response and gradually clear the infection [38, 41, 42]. The adoptive transfer of *R. equi*-specific Th1 or Th2 cells into *R. equi*-susceptible nude mice also clearly showed that the Th1 response is sufficient to affect pulmonary clearance [38]. We observed that five vaccine candidates with Freund’s adjuvant were capable of inducing a significant immune response in BALB/c mice. Two weeks after booster immunization, mice vaccinated with the vaccine candidate developed a strong antigen-specific IgG2a response. Simultaneously, the levels of the Th1 cytokines TNF-α and IFN-γ induced in mice immunized with the vaccine candidates were significantly greater than those in mice immunized with adjuvant alone. These results suggest that all five vaccine candidates can induce a Th1 cell immune response in mice, which is essential for the prevention of *R. equi* infection. In addition, immunization with the vaccine candidates produced a strong Th2 immune response. Immunization with ABC transporter, NlpC/P60, and M23 induced high levels of antigen-specific IgG1 and serum IL-4 in mice. Theoretically, Th2 responses counteract Th1-mediated intracellular bactericidal effects [39, 43, 44]. This finding is inconsistent with our hypothesis, possibly because an excessive Th1 proinflammatory response may lead to uncontrollable tissue damage. Therefore, Th1/Th2 homeostasis needs to be maintained to combat this damage [45]. Overall, these vaccine candidates have been shown to elicit antigen-specific humoral immunity and cellular immunity responses, which confirms their potential as protective antigens for the development of a vaccine against *R. equi*.

The immune protection against *R. equi* infection does not rely exclusively on the CD4 + Th1 lymphocyte response [46]. As with *M. tb*, IFN-γ-positive CD8 + T lymphocytes also contribute to immune clearance during pulmonary challenge by virulent *R. equi* [47, 48]. In this study, FCM was used to detect antigen-specific IFN-γ and TNF-α in splenic T lymphocytes, and the proportions of IFN-γ- and TNF-α-positive CD4 + and CD8 + T lymphocytes were significantly increased after immunization. This development is similar to the detection of intracytoplasmic concentrations of IFN-γ in equine bronchoalveolar lavage fluid cells, and the clearance of virulent strains is associated with an increased number of IFN-γ-producing CD4 + and CD8 + T lymphocytes in the lungs [47]. Studies have shown that rapid clearance of *R. equi* in infected horses is associated with antigen-specific proliferation of CD4 + and CD8 + pulmonary T lymphocytes [40, 47, 49]. In addition, an effective vaccine requires the induction of memory immune cells that rapidly differentiate into effector cell populations upon encountering *R. equi* and thereby eliminate pathogens [50, 51]. Our study showed that 10 weeks after immunization, splenic T lymphocytes can still rapidly release large amounts of IFN-γ and TNF-α after antigen stimulation. These results suggest that all five vaccine candidates have the potential to induce memory T lymphocyte immune responses in mice. However, this study only evaluated the memory immune response at 10 weeks post-immunization and 4 weeks after *R. equi* challenge. Further studies are needed to increase the duration of monitoring and systematically assess the ability of these agents to induce a long-term immune response. These data indicate that immunized BALB/c mice produce antigen-specific CD4 + and CD8 + T lymphocytes and memory cells, which may play a role in immunity against *R. equi*.

Immunocompetent mice can clear infected *R. equi* and usually do not experience severe pneumonia, but the organ bacterial load and pathology correlate with the degree of virulence of *R. equi* [34, 35]. Therefore, to evaluate the immunoprotective effect of vaccine candidates, we selected the lungs and livers as the organs of interest, and these organs were found to be reliable indicators for assessing the infection status of mice following *R. equi* challenge [52, 53]. One week after the *R. equi* challenge, the bacterial load (CFU) in the lungs of mice immunized with the five vaccine candidates was reduced. Two weeks after the challenge, *R. equi* was completely eliminated in the mice immunized with the NlpC/P60, Esterase, and M23 proteins. Furthermore, the NlpC/P60, Esterase, and M23 proteins successfully reduced pathological damage to the lungs and livers of vaccinated animals. In summary, all vaccine candidates protected mice against *R.equi* challenge, with the NlpC/P60, Esterase, and M23 proteins being more potent.

This study has potential limitations. Here, Freund’s adjuvant was used to prolong and augment the effect of vaccination. However, Freund’s adjuvant is often associated with the formation of painful granulomas at the vaccination site and may also result in tuberculin-type allergic reactions [54]. Due to concerns about animal welfare, researchers are exploring alternative adjuvants for use in animals. In addition, we used a BALB/c mouse infection model to evaluate the immunogenicity and immunoprotection of the vaccine candidates. Although BALB/c mice are the most widely used species in *R. equi* infection studies [34, 35], they cannot model the immunological complexity of foals and fully imitate foal *R. equi* pneumonia.

## Conclusions

In summary, ABC transporter, PBD2, NlpC/P60, Esterase, and M23 are sufficient to elicit robust antigen-specific humoral and cellular immune responses and induce protective immunity against virulent *R. equi* challenge in mice. These candidates can be used as appropriate antigens for the development of vaccines and treatments for *R. equi* infection. Further study is required to determine the ideal delivery of adjuvants, antigen combinations, and vaccines and to investigate the cellular and humoral responses elicited by the vaccine in foals exposed to *R. equi* infection.

### Electronic supplementary material

Below is the link to the electronic supplementary material.

Supplementary Material 1

Supplementary Material 2

Supplementary Material 3

Supplementary Material 4

## Data Availability

The datasets used and/or analysed during the current study are available from the corresponding author on reasonable request.
