# Supplementary material for: Evaluation of vaccine candidates against Rhodococcus equi in BALB/c mice infection model: cellular and humoral immune responses
Source: BMC Microbiol. 2024 Jul 8;24:249. doi: 10.1186/s12866-024-03408-z (PMC11229254; doi:10.1186/s12866-024-03408-z)
Supplement: Supplementary file 1 — Supplementary Material 1 [file 12866_2024_3408_MOESM1_ESM.doc]

**Survival rate of BALB/c mice after *R. equi* 103S infection**

Thirty female BALB/c mice (four-week-old) were randomly divided into five groups and mice were challenged by intraperitoneal injection (IP) with *R. equi* 103S. The challenge protocol is shown in Table S1. The symptoms and survival of mice were monitored every 12 hours (9:00 am, 9:00 pm). The results showed that all mice in the IP-4 group (5 × 10^8^ CFU) died within 12 hours after infection. All mice in the IP-3 (5 × 10^7^ CFU) group died within 6 days after infection. No mice died within 15 days of infection in the IP-2 group (5 × 10^6^ CFU) and IP-1 group (5 × 10^5^ CFU) (Fig. S1). The sublethal dose was calculated by LogLD_50_=X_K_-i(∑p-0.5), “*X_K_*” is the highest logarithmic dose, “*i*” is the difference between two adjacent logarithmic doses, and “*p*” is the mortality rate of each dose group. The results showed an LD_50_ of 2.34×10^7^ CFU for IP of *R. equi* 103S in BALB/c mice.

**Table S1 The challenge protocol for R. equi 103S in BALB/c mice**

| Infection way | Dosage  (CFU/mice) | Infection volume  (μL) | Group name | Number |
| --- | --- | --- | --- | --- |
| Intraperitoneal injection (IP) | 0-PBS | 100 | IP-control | 6 |
|  | 5×10^5^ | 100 | IP-1 | 6 |
|  | 5×10^6^ | 100 | IP-2 | 6 |
|  | 5×10^7^ | 100 | IP-3 | 6 |
|  | 5×10^8^ | 100 | IP-4 | 6 |


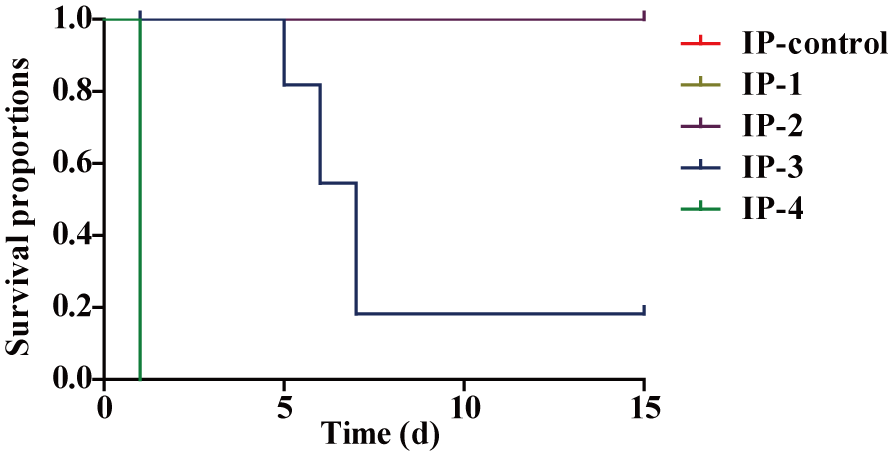


**Fig. S1** Survival rate of BALB/c mice after *R. equi* 103S infection
