# Supplementary material for: Evaluation of vaccine candidates against Rhodococcus equi in BALB/c mice infection model: cellular and humoral immune responses
Source: BMC Microbiol. 2024 Jul 8;24:249. doi: 10.1186/s12866-024-03408-z (PMC11229254; doi:10.1186/s12866-024-03408-z)
Supplement: Supplementary file 2 — Supplementary Material 2 [file 12866_2024_3408_MOESM2_ESM.doc]

## Reverse vaccinological analysis

*Screening of potential vaccine candidates*

FASTA format complete genome/proteome sequences of 16 *R. equi* strains from different countries and host sources were downloaded from the NCBI assembly database (accessed 29 march 2022) (Table S2). Use the BPGA v.1.3 tool to cluster core proteins. The subcellular localization of the core proteins was analyzed using PSORTb v3.0.2 (https://www.psort.org/psortb/index.html), CELLO v.2.5 (http://cello.life.nctu.edu.tw/), and Cell-PLoc 2.0 (http://www.csbio.sjtu.edu.cn/bioinf/Cell-PLoc-2/) servers, and membrane and extracellular proteins were selected for further analysis. Membrane and extracellular proteins were subjected to a host homology search using BLASTp (https://BLASTp.ncbi.nlm.nih.gov/BLASTp.cgi), and proteins that showed > 30% identity to host protein and a bit score > 100 were excluded. The number of transmembrane helices in the nonhost homologous proteins was predicted using the TMHMM-2.0 (https://services.healthtech.dtu.dk/service.phpTMHMM-2.0) and HMMTOP v.2.0 (http://www.enzim.hu/hmmtop/html/submit.html) tools, and proteins with < 2 transmembrane helices were selected. The antigenicity of the protein was predicted by using the AntigenPro server (http://scratch.proteomics.ics. uci.edu/index.html) that computes antigenicity based on amino acid sequence characteristics and the VaxiJen v.2.0 server (http://www.ddg-pharmfac.net/vaxijen/ VaxiJen/VaxiJen.html) that computes antigenicity based on the physicochemical properties of amino acid sequence, and the proteins with prediction scores > 0.5 were selected. The physicochemical properties of the proteins were analyzed using the Expasy ProtParam server (https://web.expasy.org/protparam/). Proteins in the MW range of 10 kDa to approximately 110 kDa were selected. Proteins with an instability index > 40 are deemed unstable and were not selected. Proteins significantly different from the host’s gut flora proteins were selected to prevent the induced immune response from having any side effects on the host. Essential proteins were subjected to a gut flora homology search using SmartBLAST. Candidate antigens that showed > 70% identity to any gut flora protein were excluded.

*Functional annotation*

To further characterize the vaccine candidates, we performed thorough manual annotations to determine the most likely biological functions of the selected vaccine candidates. We used the following annotation servers: the Cluster of Orthologous Groups (COG) (https://www.ncbi.nlm.nih.gov/research/cog/), the Kyoto Encyclopedia of Genes and Genomes (KEGG) (http://weizhongli-lab.org/metagenomic-analysis/ server), InterPro (https://www.ebi.ac.uk/interpro/), Pannzer2 (http://ekhidna2.biocenter.helsinki.fi/ sanspanz/) and eggNOG-mapper (http://eggnog-mapper.embl.de/). Furthermore, candidate antigens were subjected to a BLASTp (E-value cut off = 1e-10) with the AntigenDB (https://webs.iiitd.edu.in/raghava/ antigendb/antBLASTp.html) and Virulence Factor Database (VFDB) (http://www.mgc.ac.cn/cgi-bin/VFs/ v5/main.cgi) to identify potential virulence proteins and antigens (Table S3).

*Epitope mapping*

B-cell and T-cell epitopes are highly immunodominant elements and can stimulate significant specific immune responses. The ABCPred server (https://webs.iiitd.edu.in/raghava/abcpred/index.html) was used (threshold value > 0.8) to predict linear B-cell epitopes in the potential vaccine candidates using artificial neural networks (65.93% accuracy). The resultant 20-mer B-cell epitopes were subsequently analyzed for T-cell epitopes for binding with MHC I and MHC II alleles using the IEDB analysis resource NetMHCpan EL 4.1 (http://tools.iedb.org/mhci/) and recommended 2.22 (http://tools.iedb.org/mhcii/) tools, respectively. Epitopes with an MHC I pre-rank ≤ 0.2 and an MHC II pre-rank ≤ 10 were selected. The epitope density (ED) of each potential vaccine candidate was calculated by dividing the number of predicted epitopes by the protein length, and the potential vaccine candidates were further characterized by ED (Table S4).

Table S2 Genome/proteome information of 16 R. equi strains (NCBI-Assembly-03/29/2022 20:00)

| S/N | Strain | Host | Accession | Seq length | Date |
| --- | --- | --- | --- | --- | --- |
| 1 | 103S | *Equus caballus* | GCF_000196695.1 | 5,043,170 | 2010/11/11 |
| 2 | ATCC 33701 | *Equus caballus* | GCF_021654675.1 | 5,308,366 | 2021/11/19 |
| 3 | ATCC 33707 | *Homo sapiens* | GCF_000164155.2 | 5,259,057 | 2011/3/15 |
| 4 | DSSKP-R-001 | *soil* | GCF_003013675.1 | 5,438,826 | 2018/3/22 |
| 5 | FDAARGOS_952 | *Homo sapiens* | GCF_016025875.1 | 5,218,699 | 2020/12/14 |
| 6 | JCM94-14 | *Homo sapiens* | GCF_019703915.1 | 5,309,140 | 2021/7/12 |
| 7 | JCM94-27 | *Homo sapiens* | GCF_019703975.1 | 5,243,411 | 2021/7/12 |
| 8 | JCM94-3 | *Homo sapiens* | GCF_019704015.1 | 5,181,978 | 2021/7/12 |
| 9 | JCM94-31 | *Homo sapiens* | GCF_019703995.1 | 5,284,300 | 2021/7/12 |
| 10 | JID03-46 | *Homo sapiens* | GCF_019704055.1 | 5,358,277 | 2021/7/12 |
| 11 | JID03-56 | *Homo sapiens* | GCF_019704075.1 | 5,266,342 | 2021/7/12 |
| 12 | P2117036 | *Equus caballus* | GCF_022691685.1 | 5,142,908 | 2022/3/24 |
| 13 | P2120831 | *Equus caballus* | GCF_022691665.1 | 5,145,013 | 2022/3/24 |
| 14 | PAM2287 | *Equus caballus* | GCF_002094405.2 | 5,324,770 | 2021/9/24 |
| 15 | U19 | *Equus caballus* | GCF_022179365.1 | 5,087,417 | 2021/7/12 |
| 16 | WY | *Homo sapiens* | GCF_007197835.1 | 5,213,571 | 2019/7/21 |

**Table S3** Information about the potential vaccine candidates for *R. equi*

| Accession  number | MW (kDa) | Localisation | PredHel (TMH/HMM) | Antigenicity (VaxiJen/AntigenPro) | Instability  index | VFDB blast | AntigenDB blast |
| --- | --- | --- | --- | --- | --- | --- | --- |
| WP_013414996.1 | 57.34 | Extra | 0/1 | 0.51/0.86 | 29.16 | - | - |
| WP_013414386.1 | 51.09 | Extra/Memb | 1/1 | 0.63/0.95 | 31.42 | - | Penicillin-binding 2 *[Staphylococcus aureus]* |
| WP_013416802.1 | 38.11 | Extra | 0/0 | 0.54/0.64 | 33.54 | p60 (iap/cwhA) [*Listeria* *monocytogene*] | Protein p60  [*L.* *monocytogene*]  PPE family protein [*M.* *tb*] |
| WP_013415053.1 | 38.02 | Extra | 1/1 | 0.53/0.91 | 16.67 | Antigen 85 (FbpA/B/C) [*M.* *tb*] | Antigen 85A/B/C [*M.* *tb*] |
| WP_013415127.1 | 28.19 | Extra | 0/0 | 0.69/0.84 | 37.50 | LasA (lasA) [*Pseudomonas* *aeruginosa*] | Proline-rich 28 kDa antigen  [*M.* *tb*] |

**Table S4** The epitope density (ED) of five potential vaccine candidates

| Accession  number | One-line Annotation  (NCBI) | Length  (aa) | B-cell  ED | MHC I  ED | MHC II  ED | Overall  ED |
| --- | --- | --- | --- | --- | --- | --- |
| WP_013414996.1 | ABC transporter substrate-binding protein | 537 | 0.034 | 0.317 | 0.304 | 0.654 |
| WP_013414386.1 | Penicillin-binding protein 2 | 495 | 0.034 | 0.364 | 0.313 | 0.711 |
| WP_013416802.1 | NlpC/P60 family protein | 384 | 0.023 | 0.151 | 0.141 | 0.315 |
| WP_013415053.1 | Esterase family protein | 361 | 0.039 | 0.343 | 0.288 | 0.670 |
| WP_013415127.1 | M23 family metallopeptidase | 277 | 0.022 | 0.375 | 0.152 | 0.549 |

**Table S5** Homology of the five potential vaccine candidates in different strains

| Strain | ABC transporter | PBD2 | NlpC/P60 | Esterase | M23 |
| --- | --- | --- | --- | --- | --- |
| 103S | 100% | 100% | 100% | 100% | 100% |
| ATCC 33701 | 98.88% | 100.00% | 98.96% | 98.06% | 99.28% |
| ATCC 33707 | 99.63% | 99.80% | 98.18% | 98.89% | 99.64% |
| DSSKP-R-001 | 99.44% | 100.00% | 98.70% | 99.72% | 100.00% |
| FDAARGOS_952 | 100.00% | 100.00% | 98.96% | 98.06% | 99.28% |
| JCM94-3 | 99.81% | 100.00% | 97.66% | 99.72% | 99.64% |
| JCM94-14 | 99.44% | 100.00% | 98.96% | 98.06% | 99.64% |
| JCM94-27 | 99.63% | 100.00% | 98.96% | 100.00% | 100.00% |
| JCM94-31 | 99.81% | 100.00% | 98.70% | 100.00% | 100.00% |
| JID03-46 | 98.13% | 100.00% | 98.44% | 98.06% | 99.64% |
| JID03-56 | 98.32% | 100.00% | 98.70% | 99.72% | 99.64% |
| P2117036 | 99.63% | 100.00% | 98.96% | 99.72% | 100.00% |
| P2120831 | 99.44% | 100.00% | 98.96% | 98.61% | 99.64% |
| PAM2287 | 99.81% | 99.60% | 98.18% | 98.06% | 100.00% |
| U19 | 99.44% | 100.00% | 98.96% | 99.72% | 99.64% |
| WY | 99.44% | 100.00% | 98.96% | 99.72% | 100.00% |
