# Supplementary material for: Evaluation of vaccine candidates against Rhodococcus equi in BALB/c mice infection model: cellular and humoral immune responses
Source: BMC Microbiol. 2024 Jul 8;24:249. doi: 10.1186/s12866-024-03408-z (PMC11229254; doi:10.1186/s12866-024-03408-z)
Supplement: Supplementary file 3 — Supplementary Material 3 [file 12866_2024_3408_MOESM3_ESM.doc]

**Supplementary Sequence : Nucleotide and amino acid sequences of the five vaccine candidates**

Subclone Vaccine candidates into *E.coli* Expression Vector PET-30a(+) using NdeI and HindIII

Excluded enzyme sites: HindIII [AAGCTT]; NdeI [CATATG]

Start codon—N-His-tag-+Target protein--Stop codon

**>**[**WP_013414996.1**](https://apc01.safelinks.protection.outlook.com/?url=https://www.ncbi.nlm.nih.gov/protein/WP_013414996.1&data=05|01|tech-sunxuehua@genscript.com.cn|178aec03a72d4587ac5d08db2e7af277|dc65b2eb97864a0e9fed90b43a77c777|0|0|638154877811771265|Unknown|TWFpbGZsb3d8eyJWIjoiMC4wLjAwMDAiLCJQIjoiV2luMzIiLCJBTiI6Ik1haWwiLCJXVCI6Mn0=|3000|||&sdata=wW603c9OtQNJXmdzfBBQrzyBJS7P/M3h1wNAb4BhLko=&reserved=0) **ABC transporter substrate-binding protein** [https://www.uniprot.org/uniprotkb/E9T1Q9/entry](https://apc01.safelinks.protection.outlook.com/?url=https://www.uniprot.org/uniprotkb/E9T1Q9/entry&data=05|01|tech-sunxuehua@genscript.com.cn|178aec03a72d4587ac5d08db2e7af277|dc65b2eb97864a0e9fed90b43a77c777|0|0|638154877811771265|Unknown|TWFpbGZsb3d8eyJWIjoiMC4wLjAwMDAiLCJQIjoiV2luMzIiLCJBTiI6Ik1haWwiLCJXVCI6Mn0=|3000|||&sdata=SolI4EwzUy/WesQBoBJg0rpQQIBjsSjkEYnWVy2pGNU=&reserved=0)

**Original sequence (512 aa)**

catMHHHHHHATDATDVTTGLVGEQPDGGDAVSGGMLSYATYNAVSSLDPADRQDGGATGGTEMAAIYDVLMRYDTATKEYQPQLAQSLTPNGDNTVWTLKLRDGVKFSDGTALDAAAVQWSIDHYLEKKGTHTQVWKVTVDKVESPDPSTVVFTLKQPWNEFPIMFTTGPGMIVAPSSTANGTFTPIGAGPFTVEKFASQNELVLAANPGYWDGRPNLDKLRFPAIVGEQAKLDALHSGGIQAAYLRAADTVHNALEAGDVGYVYTASMGGVQVLNQREGRAASDPRVREAIVKALNPETFNERGEGGFGMPGTDMFQSWSQWHGDVSGSTFDPDTARKVLAEAKADGYDGKLTYAGLNEPGAQRRALAIQSMLQAVGFTVDIVYNSGINDLVKMMYAKHDFDLGESAFNVLDESPFMRMYGNLASASTSNVLGYQNPEMDALLGKLQSAPNDDDRRRVLEDIQTLVNETNPMMVLGAGKYFIPWSKNAHGITPSADGVLLFGNAWLTPDSAS*aagctt

**Optimized sequence (Average GC% - Optimized: 55.99, 1545 bp)**

catATGCATCACCACCACCACCATGCTACAGATGCGACCGATGTGACCACCGGCCTGGTGGGTGAGCAGCCGGATGGTGGCGACGCGGTTTCGGGTGGGATGCTGTCCTATGCAACGTACAACGCAGTTAGCAGCCTCGACCCGGCAGACCGCCAGGATGGCGGTGCAACCGGTGGTACGGAAATGGCAGCGATTTATGACGTTCTTATGAGGTACGACACGGCAACCAAAGAATATCAACCGCAGTTGGCTCAGTCTCTGACCCCAAATGGTGACAACACCGTTTGGACCCTGAAACTCAGAGATGGCGTGAAGTTCAGCGATGGCACGGCTCTGGACGCAGCGGCTGTGCAGTGGAGCATTGATCATTATCTGGAGAAGAAAGGTACCCATACCCAGGTATGGAAAGTCACCGTTGATAAAGTTGAATCCCCGGATCCGAGCACCGTAGTTTTCACTCTGAAGCAGCCGTGGAATGAGTTCCCGATCATGTTCACGACTGGTCCGGGTATGATTGTCGCGCCTAGCAGCACCGCGAACGGCACGTTCACCCCGATCGGCGCGGGTCCGTTCACTGTCGAGAAATTTGCATCGCAAAACGAACTGGTGTTGGCGGCGAACCCGGGCTACTGGGATGGACGTCCGAACTTAGACAAGCTGCGTTTTCCGGCAATTGTGGGCGAGCAAGCAAAGTTAGACGCACTGCACAGCGGCGGCATCCAAGCGGCGTATTTGCGCGCTGCGGACACCGTTCACAATGCCTTGGAAGCGGGTGATGTGGGTTACGTATACACCGCGTCAATGGGTGGTGTTCAAGTCTTGAACCAGCGTGAAGGTCGTGCGGCCTCCGATCCGCGTGTTCGTGAGGCCATCGTGAAGGCGCTAAACCCAGAGACATTTAACGAGCGCGGTGAAGGCGGCTTCGGCATGCCAGGTACGGATATGTTTCAGTCTTGGTCGCAATGGCATGGTGACGTGAGCGGTTCTACTTTCGACCCGGACACCGCCCGTAAAGTGCTTGCTGAAGCGAAGGCGGACGGCTACGACGGTAAGTTGACCTACGCCGGTCTGAACGAGCCGGGTGCCCAACGTCGTGCGCTTGCGATCCAAAGCATGCTGCAAGCAGTTGGTTTTACCGTGGACATCGTGTATAACAGCGGTATTAACGATCTGGTTAAAATGATGTACGCTAAACACGATTTTGACCTGGGTGAAAGCGCGTTCAACGTGCTGGACGAAAGCCCGTTTATGCGCATGTATGGTAATCTGGCGTCTGCGTCGACGTCCAATGTGTTGGGGTACCAGAATCCGGAGATGGATGCTCTGCTGGGCAAGCTGCAGTCAGCTCCGAATGATGACGACCGTCGTCGCGTGTTAGAAGATATTCAGACCCTGGTAAATGAGACGAACCCGATGATGGTTCTGGGCGCTGGCAAGTACTTCATCCCGTGGTCCAAAAACGCCCACGGCATCACCCCGAGCGCCGACGGCGTTTTGCTGTTTGGTAATGCGTGGCTGACCCCTGACAGCGCGTCCTAAaagctt

**>WP_013414386.1 Penicillin-binding protein 2**

[https://www.uniprot.org/uniprotkb/E9T837/entry#phenotypes_variants](https://apc01.safelinks.protection.outlook.com/?url=https://www.uniprot.org/uniprotkb/E9T837/entry%23phenotypes_variants&data=05|01|tech-sunxuehua@genscript.com.cn|178aec03a72d4587ac5d08db2e7af277|dc65b2eb97864a0e9fed90b43a77c777|0|0|638154877811771265|Unknown|TWFpbGZsb3d8eyJWIjoiMC4wLjAwMDAiLCJQIjoiV2luMzIiLCJBTiI6Ik1haWwiLCJXVCI6Mn0=|3000|||&sdata=jJBB8Ox9XxqC1IPenO0JP+w85AXMiSY1BkqosiscElE=&reserved=0)

**Original sequence (472 aa)**

catMHHHHHHDDLRGDPRNSRVLLDEYSRQRGQISAGGQVLASSVPTDDRYKYLRVYPPNPAAPSSPFANAPVTGFYSMQYGSAGLEKAEDPVLNGSDNRLFSKRFFDLVSGRDPRGGNVVSTIDPVMQQVAYDELTAKGYTGSVVAIEPSTGNILAMASTPSYDPNTLSSHDGAETSAAWDELNSDPDRPMLNRAVSATYPPGSTFKVVVTAAALENGANPDEQLTAAPNITLPGTSTTLENYNGTTCGGGATASLREAFARSCNTAFVELGIKTGSDALAEQAKAFGIGPGTPGIPIPVADSTIGSIPDDAALGQSSIGQRDVALTPLQNAEIAATIANGGMRMESHLVSQLQGPDLSNLATTSPKSLGRAVSPEVASTLTQLMIGSENNTAGQGKIPGVQIASKTGTAEHGNDPRNTPPHAWYIGFAPAQNPTVAIAVIVEDGGDRALAATGGSVAAPIGRAVIAAGLQRG*aagctt

**Optimized sequence (Average GC% - Optimized: 59.37, 1425 bp)**

catATGCATCACCACCACCACCATGATGACCTAAGGGGCGATCCGCGTAACTCTCGCGTGCTGCTGGATGAGTACTCTAGACAACGCGGTCAAATTAGCGCCGGTGGTCAAGTTCTGGCATCCTCCGTGCCGACCGATGACCGCTACAAATACCTCCGTGTGTATCCGCCTAACCCGGCAGCGCCAAGCTCCCCGTTTGCCAATGCACCGGTTACCGGCTTTTACAGCATGCAGTATGGTAGCGCAGGCCTGGAGAAAGCTGAAGACCCAGTACTTAATGGCTCTGACAACCGTCTGTTCAGCAAGCGCTTTTTCGATTTGGTTTCTGGTCGCGACCCGCGTGGTGGCAATGTTGTGTCCACCATCGACCCGGTTATGCAGCAGGTGGCTTACGACGAGCTCACGGCAAAGGGTTATACCGGTTCGGTTGTCGCCATTGAACCGAGCACCGGCAACATCCTGGCGATGGCAAGCACCCCGAGCTATGACCCGAACACCTTATCCAGCCATGATGGTGCGGAAACCAGCGCGGCGTGGGATGAATTAAACAGCGACCCGGACCGTCCGATGCTGAATCGTGCTGTGAGCGCAACCTACCCGCCGGGTTCCACCTTCAAGGTGGTTGTAACTGCTGCGGCGTTGGAGAACGGCGCAAATCCGGACGAGCAATTGACCGCTGCGCCAAACATCACCCTGCCGGGAACCAGTACTACGCTGGAGAACTATAATGGTACCACGTGCGGCGGTGGTGCCACGGCCTCGTTACGTGAAGCATTCGCCCGTTCATGTAATACGGCGTTCGTGGAGCTGGGCATCAAAACCGGCTCGGATGCATTGGCGGAGCAGGCAAAGGCGTTTGGTATTGGTCCGGGCACTCCGGGCATTCCGATCCCGGTTGCTGATAGCACCATCGGCTCAATTCCGGACGACGCGGCCCTGGGTCAAAGCAGTATTGGCCAGCGTGATGTCGCGTTGACCCCGCTGCAAAACGCGGAGATCGCGGCGACCATCGCGAATGGCGGCATGCGTATGGAAAGCCACCTGGTTAGCCAGCTGCAGGGTCCGGACCTCTCTAACCTGGCGACCACGTCGCCGAAATCTCTGGGTCGCGCCGTTTCGCCGGAAGTTGCGAGCACCTTGACCCAACTGATGATTGGCAGCGAAAACAACACGGCTGGTCAAGGTAAAATTCCGGGGGTGCAGATCGCTAGCAAGACCGGTACCGCGGAACATGGTAACGATCCGCGTAACACCCCGCCTCACGCATGGTACATTGGCTTCGCGCCAGCACAGAATCCGACAGTTGCTATCGCGGTCATTGTCGAGGACGGCGGCGATCGTGCTCTGGCCGCCACTGGTGGCTCCGTGGCGGCGCCCATCGGCCGGGCGGTGATCGCGGCTGGTCTGCAGCGCGGTTAAaagctt

**>WP_013416802.1 NlpC/P60 family protein**

[https://www.uniprot.org/uniprotkb/E9T333/entry](https://apc01.safelinks.protection.outlook.com/?url=https://www.uniprot.org/uniprotkb/E9T333/entry&data=05|01|tech-sunxuehua@genscript.com.cn|178aec03a72d4587ac5d08db2e7af277|dc65b2eb97864a0e9fed90b43a77c777|0|0|638154877811771265|Unknown|TWFpbGZsb3d8eyJWIjoiMC4wLjAwMDAiLCJQIjoiV2luMzIiLCJBTiI6Ik1haWwiLCJXVCI6Mn0=|3000|||&sdata=Ml25JD/YX+6mSMgPgaV5YQZ9jiqkbfbXfizYYLDNAkM=&reserved=0)

**Original sequence (391 aa)**

catMHHHHHHIDVLARPVVDLLAAFGSGALPAGSPAASLRAASEAIDAAHAVGRTGISELGGSWTGVAADAAVTKAEATQSASVQLSDRGREIATVVEAASESVRAGNAELAAILQSFLSLASSALPALATPAGQMMLIGAATEHLGRALTVVERVRGELAAHTAKIVDLTAPDPVPSDARTVAASATLPAAPVAPQVDVAGRVLSAFAGPSSGPVGSFGSSAGDVAPVAYGSGSYGSGSAVASTESGSAGGSTSYDPRFGGSGVEIVLPDGTTAVAPNEEAADAVRNALTQQGVPYQWGGTTPGQGLDCSGLTQWAYREAGVELPRLAQEQSVGVPVAQENVMPGDLAVWDGHVAMVIGNGQMVEAGDPVSISAIRTSNSGMGFHGFYRPTE*aagctt

**Optimized sequence (Average GC% - Optimized: 63.45, 1182 bp)**

catATGCATCACCATCACCACCACATAGATGTACTGGCTCGTCCGGTTGTTGACTTGCTCGCGGCGTTCGGCAGCGGTGCTCTGCCGGCAGGCTCCCCGGCGGCCTCTCTCCGTGCGGCGTCTGAAGCGATTGATGCGGCCCACGCAGTGGGTCGTACGGGCATTAGCGAGTTGGGCGGTTCATGGACTGGTGTGGCGGCGGACGCTGCCGTTACCAAGGCTGAAGCCACGCAAAGCGCAAGCGTCCAGCTGTCCGACCGTGGTCGTGAAATCGCAACCGTTGTGGAGGCGGCTAGTGAGTCTGTCAGAGCTGGCAACGCAGAACTGGCTGCGATCCTGCAGTCGTTCCTGAGCCTTGCATCCTCCGCCCTGCCTGCGCTGGCTACGCCAGCGGGTCAAATGATGCTGATCGGCGCTGCGACCGAGCACCTGGGACGTGCACTGACCGTTGTTGAGCGCGTGCGCGGTGAATTGGCGGCGCACACCGCGAAAATCGTGGACTTGACTGCGCCTGATCCGGTGCCGAGCGACGCCCGTACCGTTGCGGCCTCTGCGACCCTGCCGGCTGCGCCCGTGGCTCCGCAGGTTGATGTTGCAGGTCGCGTGCTGAGCGCGTTCGCAGGTCCGTCGAGCGGTCCGGTCGGTTCTTTTGGTTCCAGCGCGGGTGACGTGGCGCCAGTTGCCTATGGCTCAGGTTCTTACGGCTCGGGCAGCGCGGTAGCGAGCACCGAAAGCGGTAGCGCCGGCGGCAGCACCAGCTACGATCCGCGTTTCGGCGGAAGCGGTGTCGAGATCGTGCTGCCGGACGGTACTACCGCTGTGGCGCCGAATGAAGAAGCAGCGGATGCGGTGCGTAACGCCCTTACCCAACAGGGTGTCCCGTATCAGTGGGGCGGCACCACCCCGGGCCAGGGCCTGGATTGCAGCGGTCTGACGCAGTGGGCATACCGCGAGGCCGGCGTTGAGTTACCGCGCTTGGCACAAGAGCAGTCCGTGGGTGTGCCGGTGGCGCAAGAAAATGTTATGCCGGGTGATTTGGCGGTTTGGGATGGCCATGTTGCCATGGTTATTGGAAACGGCCAAATGGTGGAGGCAGGCGACCCGGTAAGCATTTCCGCTATCCGCACCTCCAACAGCGGGATGGGTTTTCATGGTTTTTATCGTCCGACGGAATAAaagctt

**>WP_013415053.1 Esterase family protein**

[https://www.uniprot.org/uniprotkb/E9T0F0/entry#ptm_processing](https://apc01.safelinks.protection.outlook.com/?url=https://www.uniprot.org/uniprotkb/E9T0F0/entry%23ptm_processing&data=05|01|tech-sunxuehua@genscript.com.cn|178aec03a72d4587ac5d08db2e7af277|dc65b2eb97864a0e9fed90b43a77c777|0|0|638154877811771265|Unknown|TWFpbGZsb3d8eyJWIjoiMC4wLjAwMDAiLCJQIjoiV2luMzIiLCJBTiI6Ik1haWwiLCJXVCI6Mn0=|3000|||&sdata=ZVmGBLII4YRyk+aEM9nmsxf9thPSshXyDT2cFM9FJ7s=&reserved=0)

**Original sequence (334 aa)**

catMHHHHHHSTPGATDAANVQASARANGGTKLVSATSTGPNKLRLVVRSESMNRDIPLDVIRPADTSKAAPTLYLLNGAGGGEDSASWQKQADIGTFFQGKQVNVVTPMQGAFTYYTDWQKADDALHGVNKWETFLTQELPPVIDSALGTTKVNSLAGISTSGTSVFNLALKDPQLYKAVGAYSGCADTVTPMGQTYIQIVIAARGSAEVENMWGPVGSPDWQAHDPIYNIGKLKGGPKLYVANASGLPGPHDTLESQGINGNVSTLANQIIVGGIIEAATNECTHRLFDAVNFNGMGDQAHFDFKPAGTHSWGYWRDDLNNSWPMLADAMGTPR*aagctt

**Optimized sequence (Average GC% - Optimized: 56.38, 1011 bp)**

catATGCATCACCACCACCACCATTCAACACCCGGCGCGACTGATGCAGCCAATGTTCAGGCCAGCGCGCGTGCAAACGGCGGCACGAAGTTGGTCTCGGCTACCTCGACCGGTCCGAATAAACTGCGTCTGGTGGTTCGTAGCGAATCTATGAATCGCGATATTCCGCTGGATGTTATTCGCCCTGCAGACACCTCCAAAGCGGCGCCAACGTTATACCTGTTGAATGGTGCTGGCGGTGGCGAGGATAGCGCGTCCTGGCAAAAACAAGCTGACATCGGCACTTTCTTCCAGGGCAAGCAAGTTAACGTGGTCACCCCGATGCAGGGCGCGTTTACCTACTACACCGATTGGCAAAAGGCCGACGACGCCCTGCATGGTGTTAACAAGTGGGAGACCTTTCTGACCCAGGAGCTGCCACCGGTGATTGACAGCGCGCTGGGTACGACCAAGGTGAATTCCCTGGCAGGCATTAGCACCTCTGGTACGAGTGTCTTTAACCTGGCGTTGAAGGACCCGCAACTGTACAAAGCGGTGGGCGCGTATAGCGGGTGCGCCGACACGGTGACCCCGATGGGTCAGACCTATATCCAGATCGTGATCGCGGCTCGTGGTAGCGCGGAAGTTGAAAACATGTGGGGTCCGGTAGGCTCACCGGATTGGCAAGCCCACGATCCGATTTATAATATTGGTAAACTTAAGGGTGGCCCGAAACTCTACGTTGCAAACGCATCCGGCCTGCCGGGTCCGCATGATACCCTTGAGAGCCAGGGTATCAACGGAAACGTGAGCACCTTGGCGAACCAGATTATCGTTGGTGGCATCATCGAGGCAGCGACCAATGAATGTACCCACCGCTTGTTCGACGCTGTGAACTTCAACGGCATGGGTGATCAGGCGCACTTTGACTTCAAACCGGCGGGTACACACAGCTGGGGTTATTGGCGTGATGACCTGAATAACAGCTGGCCGATGCTGGCGGACGCTATGGGTACGCCGCGTTAAaagctt

**>WP_013415127.1 M23 family metallopeptid ase**

[https://www.uniprot.org/uniprotkb/E9T515/entry#phenotypes_variants](https://apc01.safelinks.protection.outlook.com/?url=https://www.uniprot.org/uniprotkb/E9T515/entry%23phenotypes_variants&data=05|01|tech-sunxuehua@genscript.com.cn|178aec03a72d4587ac5d08db2e7af277|dc65b2eb97864a0e9fed90b43a77c777|0|0|638154877811771265|Unknown|TWFpbGZsb3d8eyJWIjoiMC4wLjAwMDAiLCJQIjoiV2luMzIiLCJBTiI6Ik1haWwiLCJXVCI6Mn0=|3000|||&sdata=3LbcZFZS7JCKv0oBCOhgr1LUIv6Wz/JLO9palZzooY8=&reserved=0)

**Original sequence (284 aa)**

catMHHHHHHGRHHRTSADAVLIELDPTLRRGRHREETSGPGAGLKAATVAAATGAIITAGAQLGAGSAAAAPAPAPLQVPAGLLPAGVELPQFQLPELPAAPALPTAQELIQQAKTIEIPAELPQVQDVVDNLTAAAEAAFGSVGTPNARAVKPVSGTLTSDFGPRWGSHHGGLDIAAPIGTPVYAAADGVVVDAGPASGFGLWVRVKHDDGTTTVYGHVNDYQVNVGQHVVAGQQIATVGNRGQSTGPHLHFEVWSPGGAKIDPSSWLEERGVAVTWRGASATM*aagctt

**Optimized sequence (Average GC% - Optimized: 62.72, 861 bp)**

catATGCATCACCACCACCATCACGGAAGGCACCACCGCACCTCTGCCGATGCCGTCCTGATTGAGCTCGACCCGACCCTGCGTCGTGGTCGTCATAGAGAAGAAACCTCCGGCCCGGGTGCGGGCCTGAAAGCAGCGACGGTGGCTGCGGCGACCGGTGCAATTATCACCGCAGGCGCGCAGCTGGGTGCGGGCTCTGCAGCTGCGGCACCGGCGCCAGCTCCGCTGCAGGTCCCGGCGGGGTTGCTGCCAGCGGGCGTCGAGCTGCCGCAGTTCCAGTTACCGGAACTTCCGGCTGCGCCTGCTCTGCCGACTGCGCAAGAGTTGATCCAGCAAGCAAAAACCATTGAAATTCCGGCGGAGTTGCCGCAGGTGCAGGATGTTGTTGACAACCTGACCGCGGCCGCGGAAGCAGCGTTTGGCAGCGTTGGAACTCCGAACGCACGTGCAGTGAAACCGGTTAGCGGTACGTTGACCTCGGATTTCGGCCCTCGTTGGGGTTCCCATCATGGTGGTCTGGACATCGCCGCGCCCATCGGCACCCCGGTTTATGCTGCTGCCGACGGCGTGGTGGTAGACGCAGGTCCGGCTAGCGGTTTTGGTCTGTGGGTTCGTGTTAAGCACGATGATGGTACAACCACCGTGTACGGCCACGTGAACGACTACCAGGTTAATGTTGGCCAACACGTGGTAGCCGGCCAACAAATTGCAACGGTGGGCAATCGCGGTCAAAGCACCGGTCCGCATCTGCACTTCGAGGTTTGGTCACCGGGTGGTGCCAAGATCGATCCGAGCAGCTGGCTGGAAGAGCGCGGTGTGGCGGTGACCTGGCGTGGCGCGAGCGCGACGATGTAAaagctt
