# Supplementary material for: Evaluation of vaccine candidates against Rhodococcus equi in BALB/c mice infection model: cellular and humoral immune responses
Source: BMC Microbiol. 2024 Jul 8;24:249. doi: 10.1186/s12866-024-03408-z (PMC11229254; doi:10.1186/s12866-024-03408-z)
Supplement: Supplementary file 4 — Supplementary Material 4 [file 12866_2024_3408_MOESM4_ESM.doc]

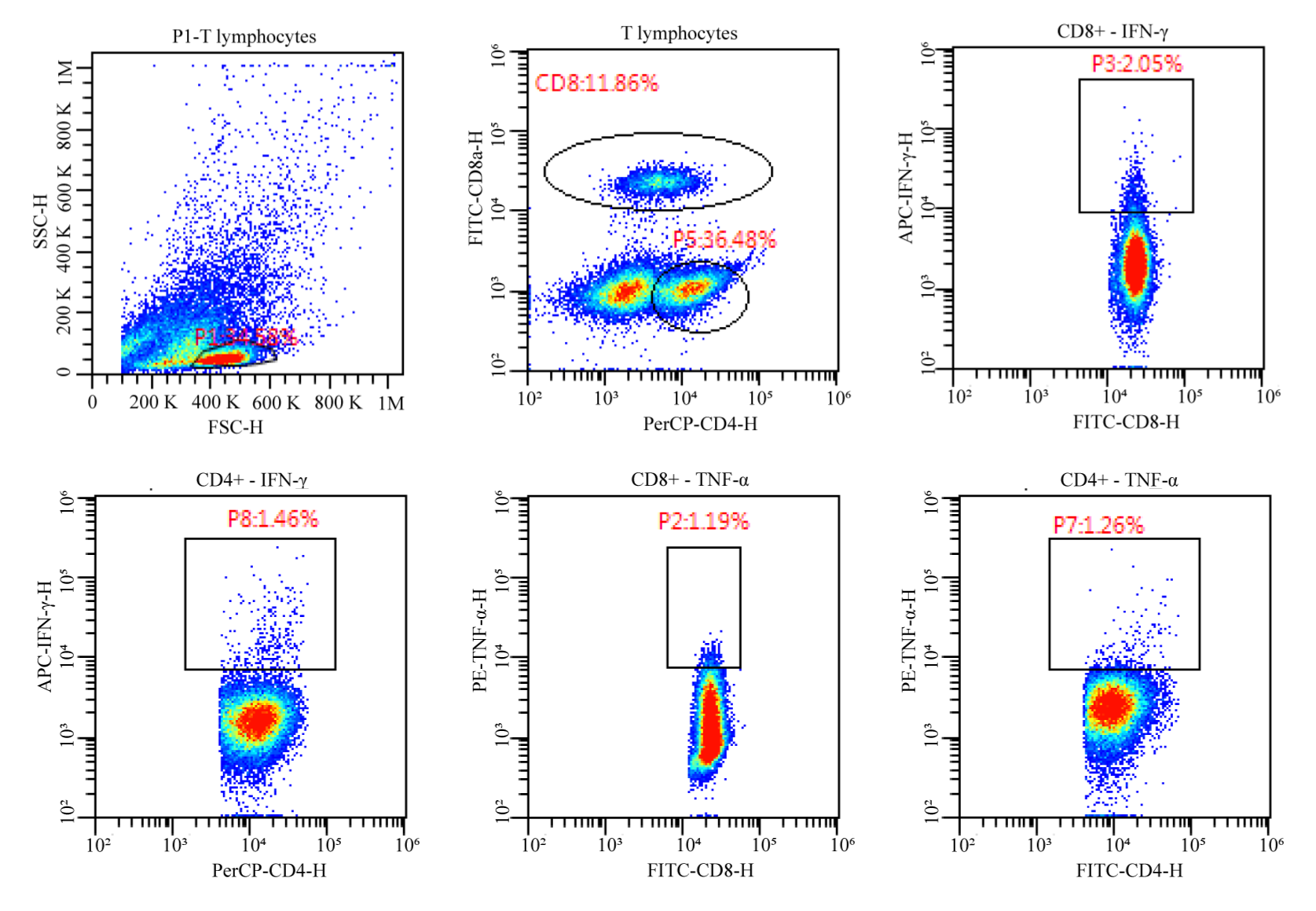


**Fig. S2 Gating strategy for the intracellular staining assay.** Two weeks after the last immunization and 1, 2, and 4 weeks post-challenge, mice were euthanized; their spleens removed; single cell suspensions prepared and stimulated 16 h in T-cell medium with individual ABC transporter, PBD2, NlpC/P60, Esterase, and M23 protein. Then, Brefeldin A (10 μg/mL, 423303, Biolegend, California, USA) diluted in T-cell medium was added to all wells and incubated with cells for an additional 6 h. Following in vitro stimulation, cells were harvested; washed with Staining Buffer (MB-089-0500, Rockland, Limerick, Ireland), and then incubated with PerCP Anti-Mouse CD4 (100431, Biolegend) and FITC Anti-Mouse CD8a (100803, Biolegend) antibody for 30 min at 4  °C in dark. Afterwards, cells were fixed and permeabilized with FIX&PERM solution kit (GAS-002M, Nordic Mubio) for 20 min, and washed with PERM wash buffer. Then, cells were stained with APC Anti-Mouse IFN-γ (505809, Biolegend) and PE Anti-Mouse TNF-α (506305, Biolegend) at 4 °C for 30 min, shaded from the light. Cells were washed twice with Perm Wash buffer, resuspended in staining buffer, visualized and analyzed using Flow Cytometry (S3e, Bio-Rad, California, USA), a minimum of 60,000 stained cells per sample was acquired. Initial gating of total events included a lymphocyte gate, followed by selection CD4+ and CD8+ T cells. The gates for frequencies of antigen-specific IFN-γ and TNF-α producing CD4+ and CD8+ T cells were determined using the unstimulated cells. Frequencies of each cell population are shown within each plot.
